# Protective effects of Pudilan Tablets against osteoarthritis in mice induced by monosodium iodoacetate

**DOI:** 10.1038/s41598-023-29976-0

**Published:** 2023-02-16

**Authors:** Zhizheng Fang, Xiangyu Li, Shujun Lei, Shibin Feng, Chenyu Zhou, Xiaohui Tong, Rongchun Han

**Affiliations:** 1 School of Pharmacy, Anhui University of Chinese Medicine, Hefei, 230012 China; 2 Department of Research and Development, Anhui Jiren Pharmaceutical Company, Bozhou, 236800 China; 3 College of Animal Science and Technology, Anhui Agricultural University, Hefei, 230036 China; 4 School of Life Sciences, Anhui University of Chinese Medicine, Hefei, 230012 China

**Keywords:** Pharmacology, Interleukins, Tumour-necrosis factors

## Abstract

Osteoarthritis (OA) is a complicated disorder that is the most prevalent chronic degenerative joint disease nowadays. Pudilan Tablets (PDL) is a prominent traditional Chinese medicine formula used in clinical settings to treat chronic inflammatory illnesses. However, there is currently minimal fundamental research on PDL in the therapy of joint diseases. As a result, this study looked at the anti-inflammatory and anti-OA properties of PDL in vitro and in vivo, as well as the mechanism of PDL in the treatment of OA. We investigated the anti-OA properties of PDL in OA mice that were generated by monosodium iodoacetate (MIA). All animals were administered PDL (2 g/kg or 4 g/kg) or the positive control drug, indomethacin (150 mg/kg), once daily for a total of 28 days starting on the day of MIA injection. The CCK-8 assay was used to test the vitality of PDL-treated RAW264.7 cells in vitro. RAW264.7 cells that had been activated with lipopolysaccharide (LPS) were used to assess the anti-inflammatory properties of PDL. In the MIA-induced OA model mice, PDL reduced pain, decreased OA-induced cartilage damages and degradation, decreased production of pro-inflammatory cytokines in serum, and suppressed *IL-1β*, *IL-6*, and *TNF-α* mRNA expression levels in tibiofemoral joint. In RAW264.7 cells, PDL treatment prevented LPS-induced activation of the ERK/Akt signaling pathway and significantly decreased the levels of inflammatory cytokines, such as IL-1β, IL-6, and TNF-α. In conclusion, these results suggest that PDL is involved in combating the development and progression of OA, exerts a powerful anti-inflammatory effect on the knee joint, and may be a promising candidate for the treatment of OA.

## Introduction

Osteoarthritis (OA), the most common degenerative joint disease characterized by chronic pain and joint impairment, is caused by articular cartilage loss and inflammatory mediator-driven joint tissue healing^1–3^. The most frequent type of OA involves knee joint featuring cartilage disintegration and bone responses around joints^4^. With the growth in obesity rates and the aging of the population, OA is now ubiquitous globally, which affects over 240 million individuals worldwide and has become the most common cause of mobility restriction in adults^5^. Patients have suffered major health repercussions, as well as physical and psychological distress, as a result of OA^6^. Treatment can be broadly divided into non-drug therapy, drug therapy, intra-articular injection therapy, and surgical therapy^7^.

OA was traditionally thought to be a non-inflammatory joint disease due to its cartilage degeneration, but new researches reveal that inflammation plays a crucial role in the etiology of OA, triggering several pathological changes^8^. Concentrations of certain inflammatory cytokines such as interleukin 1beta (IL-1β), interleukin 6 (IL-6) and tumor necrosis factor alpha (TNF-α) were found to be significantly higher in the knee joints of OA patients^9^. Furthermore, studies have revealed that inflammation not only contributes to the incidence and progression of OA, but also mediates the genesis of pain^10^. With macrophages infiltrating synovial membranes and activated macrophages mediated through mechanistic target of rapamycin (mTOR), nuclear factor-kappaB (NF-κB), c-Jun N-terminal kinase (JNK), phosphoinositide 3-kinase (PI3K), protein kinase B (Akt), and other signaling pathways, inflammation has a substantial influence on OA. Elevated levels of pro-inflammatory cytokines in blood and synovial fluid samples in vivo, as well as increased release of inflammatory mediators (including IL-1β, IL-6, and TNF-α), cause the expression of matrix metalloproteinases and other protein hydrolases, resulting in cartilage fracture^11,12^.

There are many ways to prepare OA animal models. Chemically induced models are preferred because they avoid post-surgery complications^13^. The compound injected into the knee joint in various researches is mostly monosodium iodoacetate (MIA), which inhibits the activity of glyceraldehyde-3-phosphate dehydrogenase in the glycolytic pathway, causing disruption of glycolytic energy metabolism and ultimately the death of articular cartilage cells^14,15^. This model is widely used to study the histological changes caused by OA^16^. Although MIA-induced OA cannot fully mimic the process of primary OA in humans, the articular histological changes are similar to those in human, namely, synovitis and cartilage degeneration^17–19^. In addition, the occurrence, progression, and severity of OA can be controlled by changing the dose of MIA, thus helping to study different grades of OA^20,21^.

To our best knowledge, PDL has not been shown to treat OA in rodent models. Given the potent effects of PDL on treating inflammatory diseases as well as the fact that the underlining mechanisms of OA involve inflammation^22–24^, we set out to assess of OA, mechanical hypersensitivity test (von Frey test) was utilized to analyze whether PDL could relieve pian caused by injection of MIA into articular cavity. Moreover, to determine the involvement of PDL in the development of OA, our study focused on the mechanisms that PDL reduced the inflammatory response and protective effects against cartilage damage in OA. We interrogated the anti-inflammatory and anti-OA effects of PDL against OA in both in vitro model utilizing RAW 264.7 cells treated with LPS, as well as in vivo experiments adopting the MIA-induced OA mouse model.

## Results

### The UPLC chromatograms of PDL

**Figure 1:**
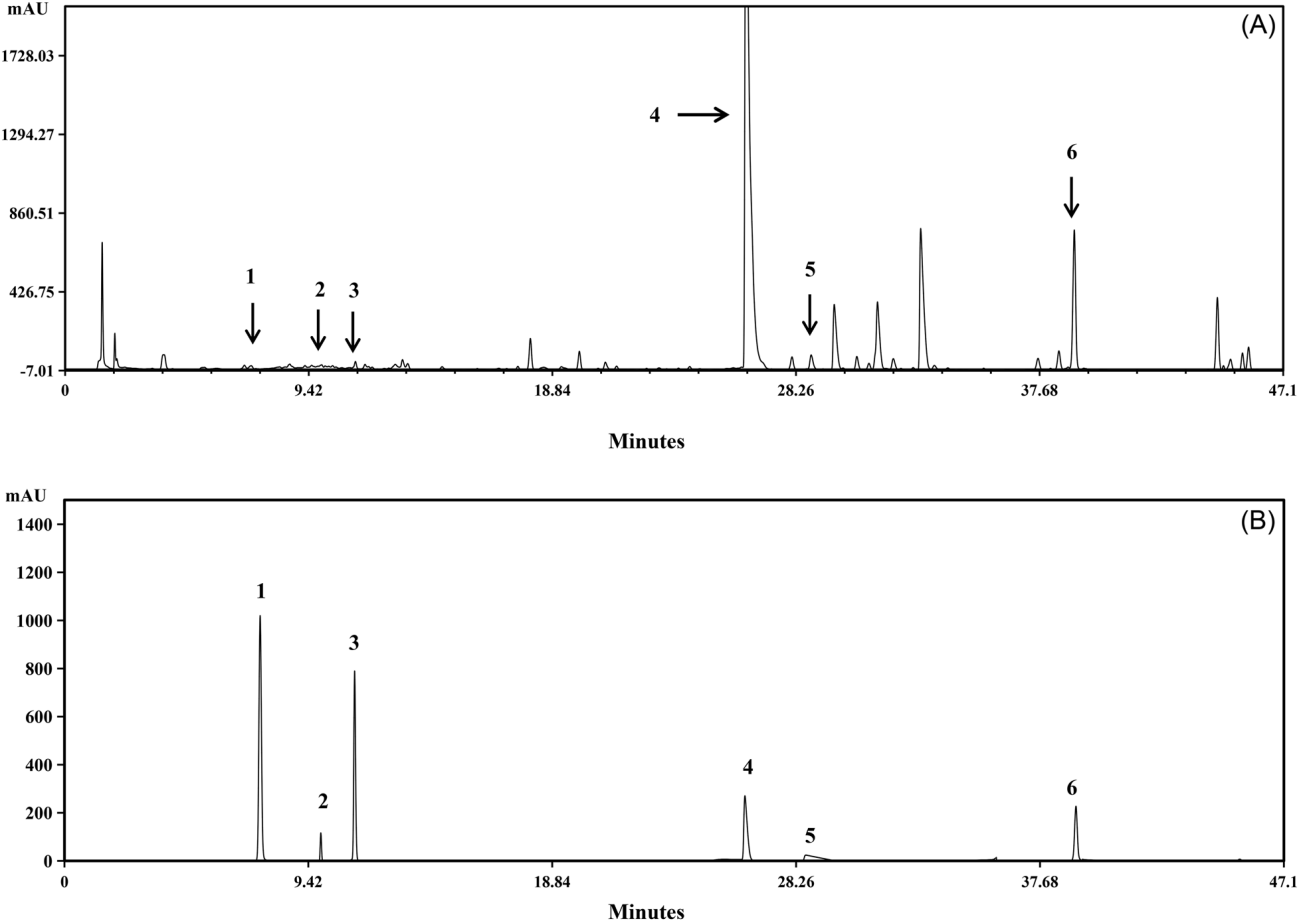
The traditional Chinese medicine system of evaluating fingerprint similarity was used to create a representative Ultra-Performance Liquid Chromatography chromatogram of Pudilan at 289 nm (**A**) and the reference chromatogram (**B**). The six main peaks were represented by the peaks numbered 1–6 in the chromatogram. The peaks denoted by the numbers 1, 2, 3, 4, 5 and 6 were, respectively, epigoitrin, chlorogenic acid, caffeic acid, baicalin, acetylcorynoline and baicalein.

PDL is a traditional Chinese medicine formula composed of four kinds of Chinese herbal medicines that contain a variety of biologically active ingredients**.** The standard curve was established by integrating the chromatograms of the standard substances. The contents of six characteristic components in Pudilan Tablets, namely, epigoitrin, chlorogenic acid, caffeic acid, baicalin, acetylcorynoline, and baicalein, were determined by the ultra-performance liquid chromatograph (UPLC) system (Agilent 1290, USA). Our established UPLC method has the characteristics of rapidity, accuracy, and stability that can provide a reference for quality control and in-depth pharmacological studies of PDL (Fig. 1).

### Effects of PDL on body weight and mechanical hypersensitivity post MIA injection

**Figure 2:**
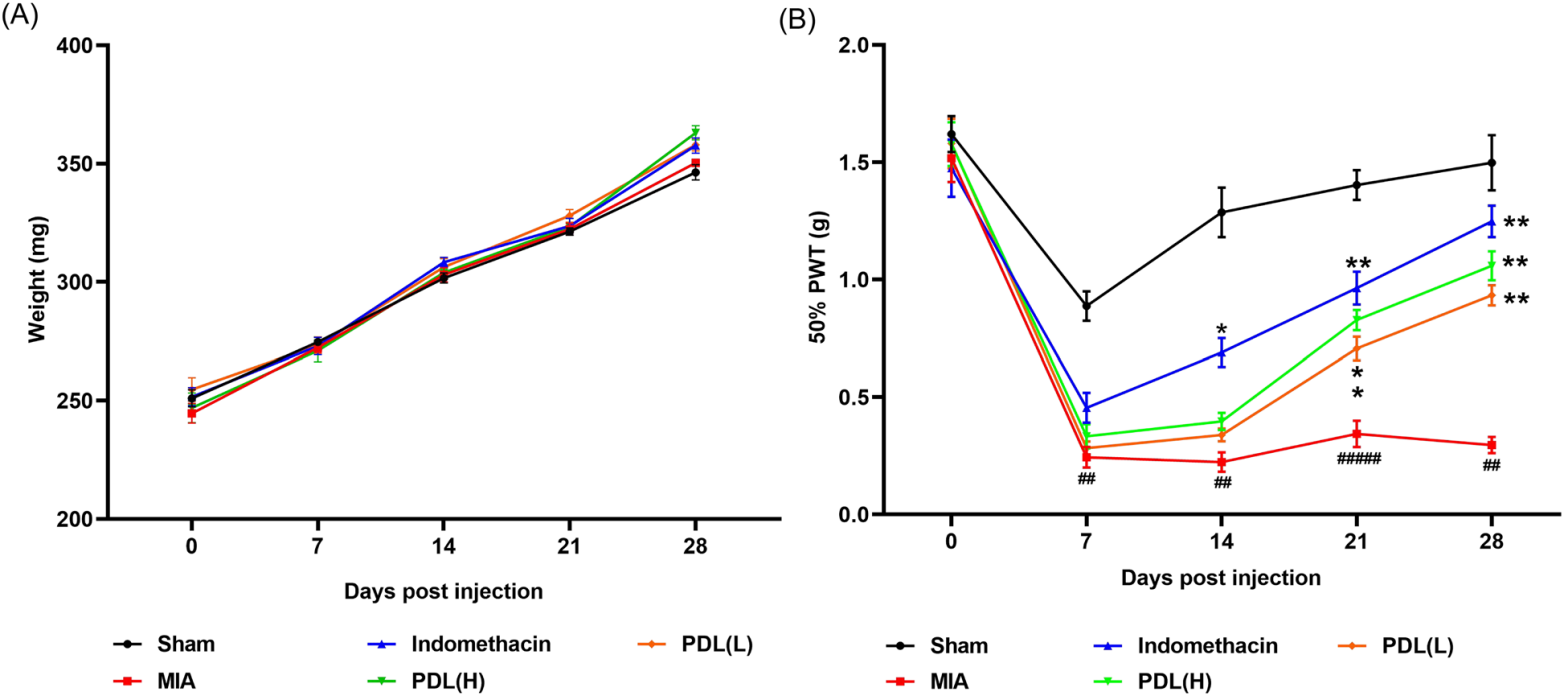
Effect of PDL treatment on body weight and mechanical hypersensitivity in monosodium iodoacetate (MIA)-induced OA mice. (**A**) The effect of PDL treatment on the mouse body weight. (**B**) The effect of PDL treatment on mechanical hypersensitivity in mice. All data were shown as mean ± SD (n = 10, ##p < 0.01, #####p < 0.0001 compared to the Sham group. *p < 0.05, **p < 0.01 compared to the MIA group).

As shown in Fig. 2A, PDL did not significantly alter the upward trend in body weight over 28 days. No significant change in body weight was observed in either the PDL treated or OA mice compared to the Sham group. This indicates that PDL treatment and MIA injection have no effect on the body weight of the subjects. The most prevalent symptom of OA is joint pain. Early on, the feeling of pain could be mild, but as the disease progresses, the pain symptoms will become increasingly severe. The von Frey test is a non-invasive approach for evaluating mechanical hypersensitivity in mice and rats. The von Frey test was used to determine the effect of PDL on pain in MIA-induced mice (Fig. 2B). Overall, on the last day, the 50% paw withdrawal threshold (PWT) in the MIA group was significantly lower compared to the group of sham (0.296 ± 0.07 vs 1.498 ± 0.264). Meanwhile, medications slowed or even reversed the pain progression of OA in comparison to MIA group (PDL-H: 1.058 ± 0.138, PDL-L: 0.933 ± 0.096 vs 0.296 ± 0.07). In more detail, one week after MIA injection, the 50% PWT value declined rapidly (0.244 ± 0.097 vs 0.887 ± 0.139), with a significant difference, demonstrating that the MIA-induced OA model was successfully established. On the 14th day, although the 50% PWT of the PDL treatment group (PDL-H: 0.397 ± 0.082, PDL-L: 0.338 ± 0.061 vs 0.224 ± 0.092) was somewhat higher than the MIA group, there was no significant difference between the two groups. When compared to the MIA group, the value of the treatment group (PDL-H: 0.827 ± 0.095, PDL-L: 0.706 ± 0.112 vs 0.343 ± 0.126) improved significantly by the third week, and the medicine reversed the OA process. At the end of the fourth week, 50% PWT in the therapy group (PDL-H: 1.058 ± 0.138, PDL-L: 0.933 ± 0.096 vs 0.296 ± 0.077) had recovered to levels higher than the prior weeks. As a result, PDL exhibits considerable analgesic effect compared with the model group, although given the dose and treatment time in this experiment, it fails to restore the pain threshold of mice to Sham group.

### Effect of PDL treatment on the histopathology of the knee joint in mice with OA

**Figure 3:**
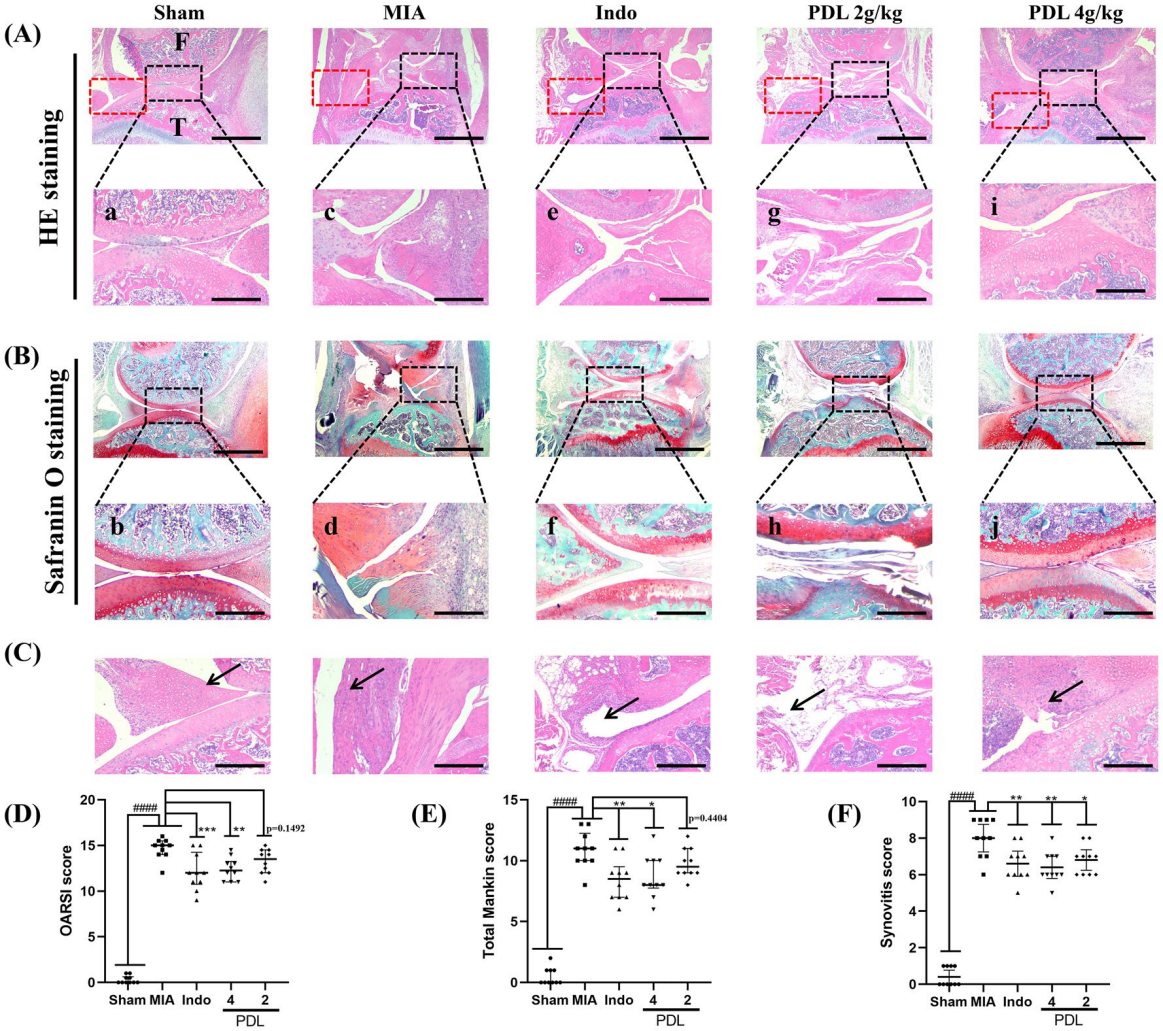
Effect of PDL on MIA-induced pathological changes on the tibiofemoral articular cartilage of the medial tibiofemoral joint. (**A**) Histopathology (H&E stain) of sagittal section (a, c, e, g, i) of tibiofemoral articular cartilage, with femoral condyle at the top and tibial plateau at the bottom. T = tibia. F = femur. Scale bars = 500 µm (upper row), 100 µm (lower row). (**B**) Chondrocytes and proteoglycan layer of tibiofemoral joint stained with Safranin O (b, d, f, h, j). Scale bars = 500 µm (upper row), 100 µm (lower row). (**C**) The synovial membrane with H&E staining from the red dotted box area in (**A**) is shown, and the black arrow indicates the synovial surface. Scale bars = 100 µm. H&E and Safranin O staining sections from each group was used for the assessment of PDL via (**D**) OARSI score, (**E**) modified Mankin score, and (**F**) Severity of synovitis evaluated by Synovotis scores. The data were present as the median with interquartile range. (Two observers contributed scores, n = 5, ####p < 0.0001 compared with the Sham group. *p < 0.05, **p < 0.01, ***p < 0.005 compared with the MIA group).

The knee joint tissue sections of mice were stained with hematoxylin and eosin (H&E) and Safranin O/Fast green to observe the pathological changes in the tibiofemoral articular cartilage of the medial tibiofemoral joint in each group (Fig. 3A,B). The results showed that the cartilage structure and tide line of the mice in the sham group were intact, and the distribution of chondrocytes was uniform and regular. The cartilage edge of the mice in the MIA group was uneven and severely eroded with the number of chondrocytes reduced and the thickness of the cartilage layer thinned, and the Safranin O staining reduced or even disappeared. Both PDL and indomethacin (Indo) administration could reduce cartilage layer damage to varying degrees, restore chondrocyte numbers, and improve cartilage layer structure. In addition, we observed H&E sections to assess synovial inflammation in all groups (Fig. 3C). In the MIA group, certain deteriorating histological changes in the synovium were observed, including an increased surface lining cell layer, high synovial cell density, surface inflammatory infiltration, and mild inflammatory infiltration into the infrapatellar fat pad. The PDL group had neatly arranged synovial lining cells, sparse connective tissue, and less inflammatory cell infiltration than the MIA group (Fig. 3C). Furthermore, the Indo and PDL treatment groups had lower Osteoarthritis Research Society International (OARSI) scores (Fig. 3D) and modified Mankin scores (Fig. 3E) than the MIA group. The results of the synovitis scores (Fig. 3F) were significant, with lower scores in the PDL group than in the MIA group.

### Effect of PDL treatment on radiographic changes in a MIA-induced OA model

**Figure 4:**
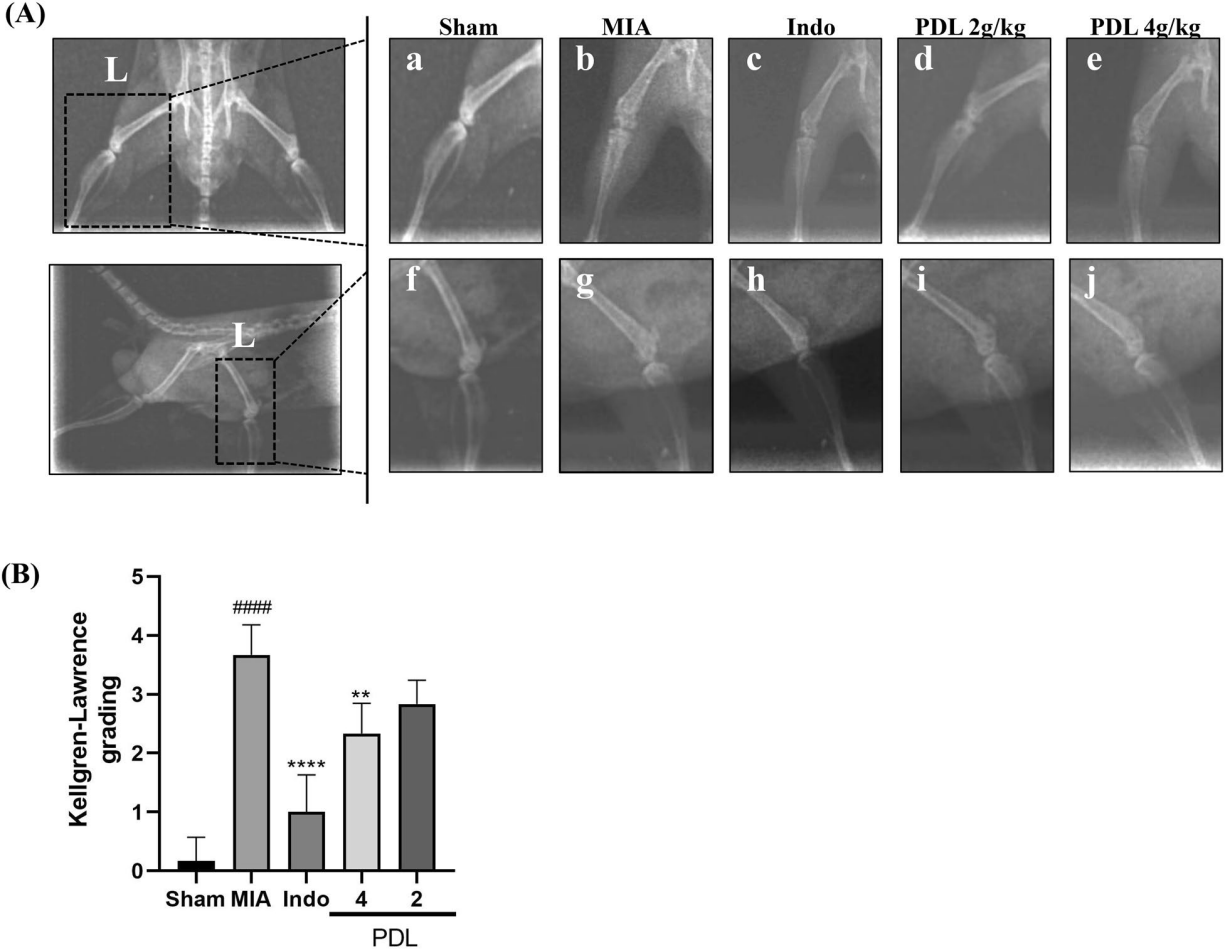
Effects of PDL as quantitatively assessed by X-ray imaging in the MIA induced OA mice model. (**A**) Representative radiographs of the left knee (L) in the sham (sham), sodium iodoacetate (MIA), MIA + indomethacin and PDL groups after day 28 of treatment. Anteroposterior (a, b, c, d, and e) and lateral (f, g, h, i, and j) radiographs of the knee joint in mice. (**B**) Results of KL grade scoring for each group. The data were present as mean ± SD. (n = 5, ####p < 0.0001 compared with the Sham group. **p < 0.01, ****p < 0.0001 compared with the MIA group).

At the end of the treatment period, the anteroposterior and lateral views of the left knee joint in all groups were X-rayed to assess the damage to the articular cartilage surface (Fig. 4A). X-ray results showed that the knee joint of the mice in the Sham group had a regular bone profile, no hyperplasia, no deformation, no narrowing of the knee joint space, and no visible bone redundancy. Compared with the sham group, the knee joint of the MIA group showed OA changes, a significant narrowing of the joint space, and osteophytes and osteosclerosis formation. In contrast, the PDL-treated knees showed significant restoration of normal joint morphology, marked by restoration of decreased joint space and reduced osteophytes. Our results suggest that PDL has a good ameliorative effect on MIA-induced OA. Assessment of X-ray imaging according to the Kellgren-Lawrence (K-L) classification showed significantly reduced scores in the PDL-treated (4 g/kg) group (vs MIA, p < 0.01), as shown in Fig. 4B.

### PDL reducing the mRNA expression of IL-1β, IL-6 and TNF-α in tibiofemoral joint

**Figure 5:**
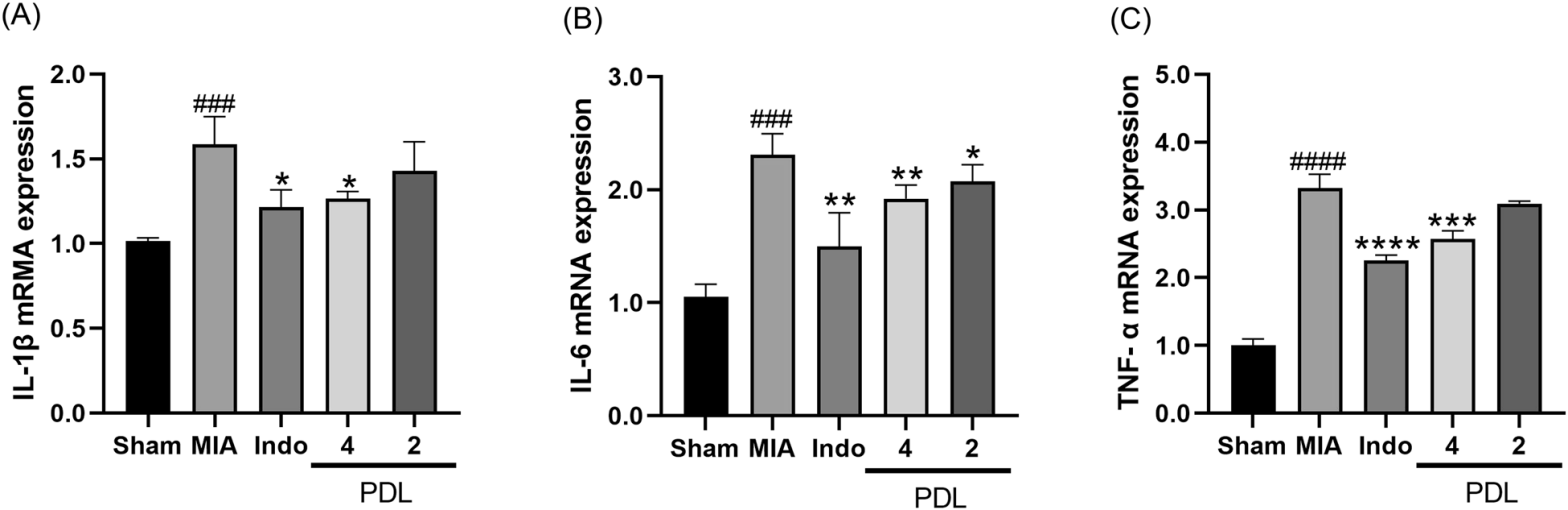
The effect of PDL on mRNA levels of IL-1 (**A**), IL-6 (**B**), and TNF- (**C**) in tibiofemoral joint in MIA-induced OA mice. Data were presented as mean ± SD. (n = 5, ^###^p < 0.005, ^####^p < 0.0001, compared with the Sham group. *p < 0.05, **p < 0.01, ****p < 0.0001, compared with the MIA group).

To explore the role of PDL on articular cartilage integrity mechanisms, we performed quantitative real time PCR (qPCR) to examine the effects of PDL on the expression of inflammatory mediators and cytokines (*i.e., IL-1β*, *IL-6*, and *TNF-α*) in knee cartilage tissue. The qPCR results revealed that the MIA group had significantly higher relative mRNA levels of *IL-1β* (^###^p < 0.005), IL-6 ^(###^p < 0.005) and *TNF-α* (^####^p < 0.0001) than the sham group (Fig. 5). PDL treatment significantly reduced the mRNA levels of *IL-6* in the articular cartilage of MIA-induced OA mice compared to those of the model mice (Fig. 5A). However, in terms of suppressing *TNF-α* and *IL-1β* expression, the medium dose of PDL was not as effective as the high dose. For *TNF-α* and *IL-1β*, high doses of PDL significantly reduced expression levels compared to the MIA group, however, the data did not show a significant reducing effect of medium doses of PDL (Fig. 5B,C).

### PDL reducing MIA-induced OA inflammation by lowering the levels of pro-inflammatory cytokines IL-1β, IL-6, and TNF-α

**Figure 6:**
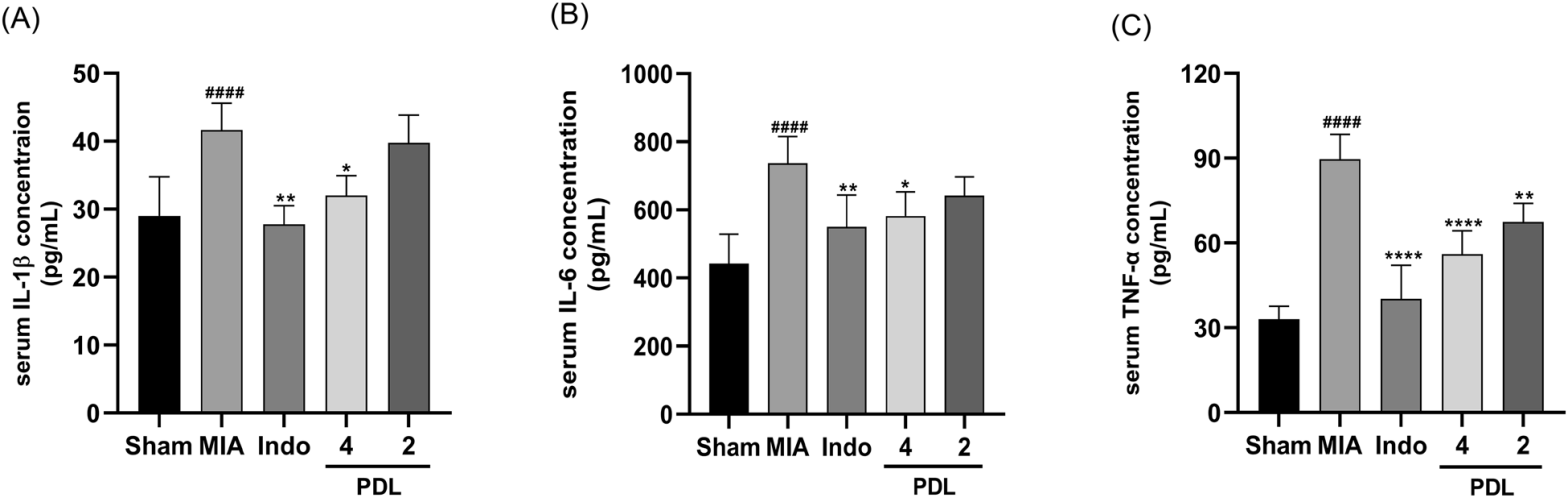
PDL presented anti-inflammatory activities in OA mice. After 4 weeks treatment, the serum IL-1β (**A**), IL-6 (B) and TNF-α (**C**) levels were examined by ELISA. Data were presented as mean ± SD. (n = 5, ^####^p < 0.0001, compared with the Sham group. *p < 0.05, **p < 0.01, ****p < 0.0001, compared with the MIA group).

We measured serum levels of inflammatory cytokines to check if PDL affects the production of pro-inflammatory cytokines and the effect of PDL on MIA-induced inflammation in OA mice. Enzyme-linked immunosorbent assay (ELISA) results revealed that IL-1β, IL-6, and TNF-α levels were significantly elevated in the serum of MIA-induced OA mice, whereas PDL administration counteracted the elevated levels of above-mentioned cytokines induced by MIA injection, as shown in Fig. 6. PDL at a dose of 4 mg/kg significantly reduced serum levels of IL-1β, IL-6, and TNF-α in OA mice compared to MIA-induced OA mice. Our ELISA results show that PDL had anti-inflammatory effects in OA mice.

### PDL suppressing the inflammatory response in LPS-stimulated RAW264.7 cells

**Figure 7:**
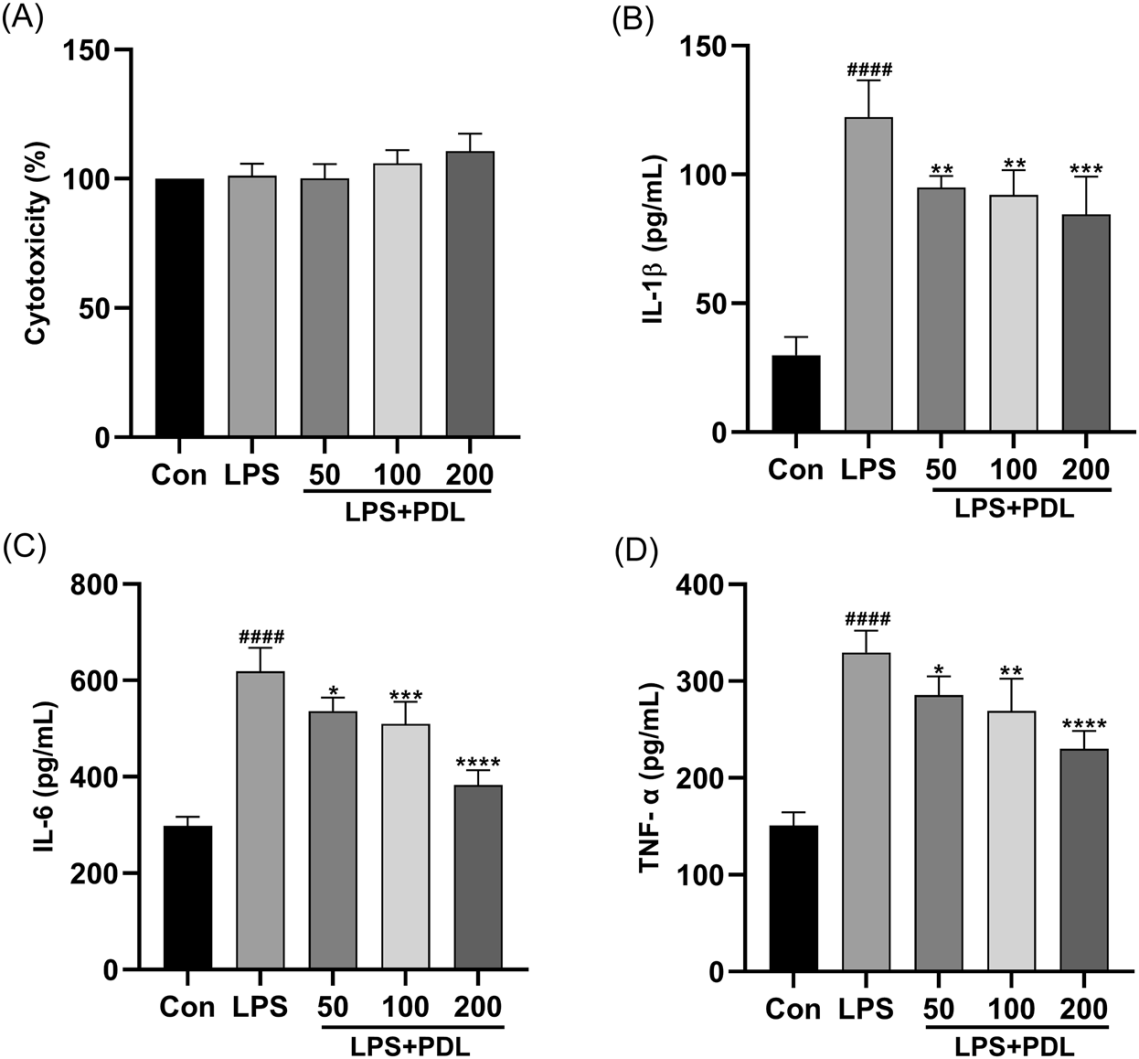
PDL inhibited the inflammatory response of LPS-induced RAW264.7 cells. Effect of PDL on (**A**) cytotoxicity, (**B**) IL-1β, (**C**) IL-6 and (**D**) TNF-α production in RAW264.7 macrophages activated with LPS. The values are expressed as the mean standard deviation (n = 3). ^####^p < 0.0001 vs vehicle control cells. *p < 0.05, **p < 0.01, ***p < 0.001, ****p < 0.0001 vs LPS-treated cells.

LPS-stimulated RAW264.7 cells were treated with PDL to test how it affected the inflammatory response. PDL had no effect on the viability of RAW264.7 cells, and the data revealed no cell toxicity (Fig. 7A). The effect of PDL on the inflammatory response was assessed following LPS stimulation of RAW264.7 cells (Fig. 7B–D). In LPS-treated RAW264.7 macrophages, LPS significantly increased IL-1β, IL-6 and TNF-α production in the cells when compared to the control group. PDL treatment, on the other hand, reduced the level of IL-1β, IL-6 and TNF-α stimulated by LPS in a dose-dependent manner. Compared with two other dosages tested, PDL at a dose of 200 μg/mL demonstrated best effect suppressing production of IL-1β, IL-6, and TNF-α induced by LPS.

### PDL inhibiting LPS-induced ERK/Akt pathway activity in RAW264.7 cells

**Figure 8:**
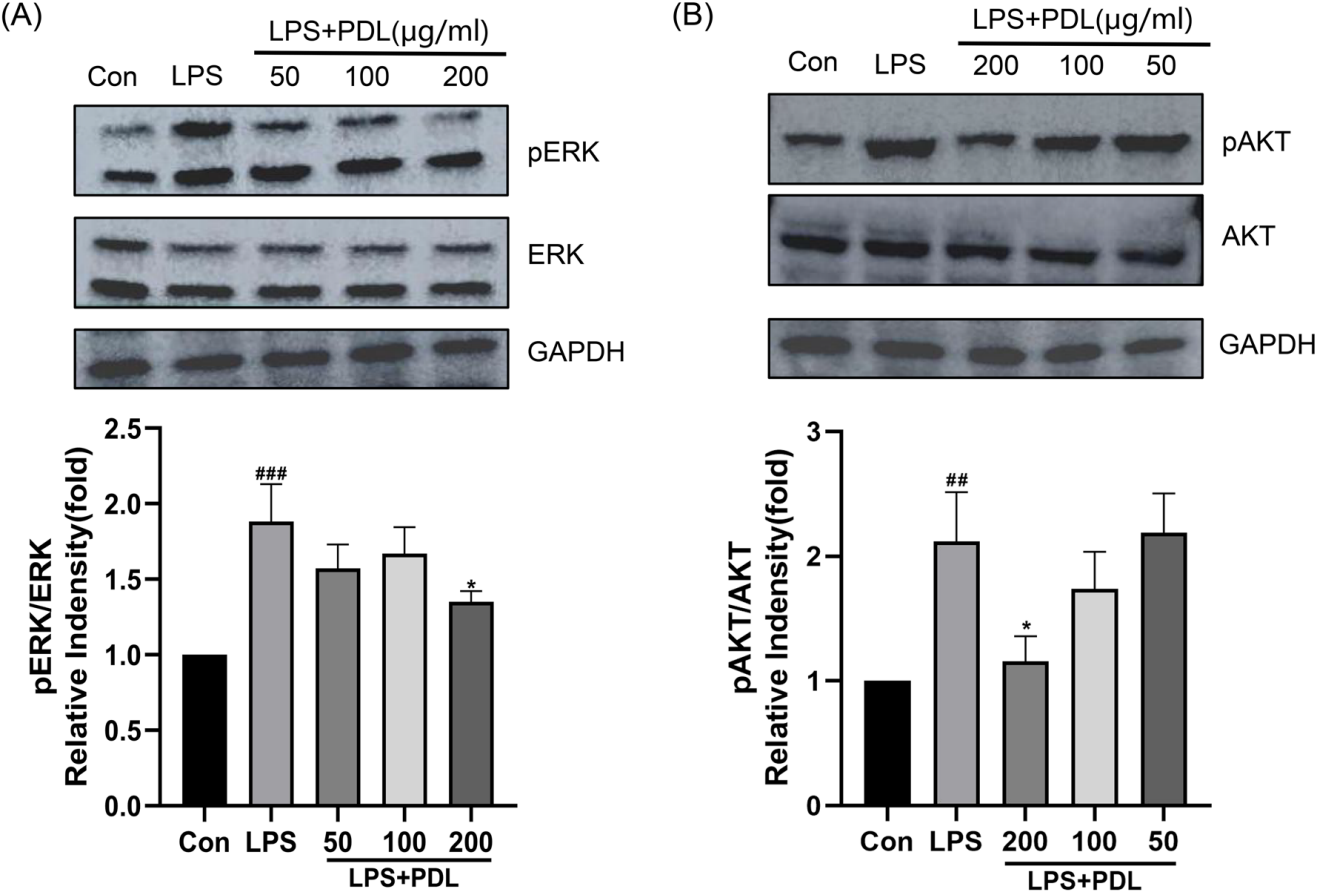
Effect of PDL on ERK/Akt phosphorylation in LPS-induced RAW 264.7 cells. The protein expressions of (**A**) ERK and (**B**) Akt proteins were visualized by Western blotting analysis. Quantitative analysis of each protein by image J. The values of each control were used to estimate the levels of ERK and Akt. The values are shown as the mean ± SD (n = 3). ^##^p < 0.01, ^###^p < 0.005 vs vehicle control cells. *p < 0.05 vs LPS-treated cells.

The molecular mechanism of the effect of PDL on the signaling pathway of ERK/Akt, which is upstream to the subsequent inflammatory response, in LPS-treated RAW264.7 cells was probed by Western blotting assay to detect ERK/Akt protein phosphorylation levels. As shown in Fig. 8, the expression levels of Akt and ERK phosphorylated proteins were highly significantly upregulated in the LPS group compared with the control group (p < 0.01), while the expression levels of Akt and ERK phosphorylated proteins were significantly downregulated in the PDL (200 μg/mL) groups compared with that of the LPS group (p < 0.05). Although the expression levels of ERK/Akt phosphorylated proteins were downregulated in the PDL (50/100 μg/mL) group compared to the LPS group, no significant difference was observed (p > 0.05).

## Discussion

OA is a complicated degenerative disease that causes patients to experience pain, stiffness, and functional restrictions, resulting in a lower quality of life^25^. Unfortunately, there is no effective cure for OA, and current medical therapies can relieve discomfort and provide some respite, but they do not stop the illness from progressing^26^. Currently, the mainstream opinion on mechanism of OA pathogenesis involves biomechanical damage leading to mediators release, which results in the activation of multiple inflammatory pathways that break down the cartilage^27^. Therefore, OA treatment focusing on inflammatory responses as well as reducing pain and swelling is a practical strategy.

Common OA treatments, such as acetaminophen, nonsteroidal anti-inflammatory drugs (NSAIDs), and opioids, have not been updated in recent decades, despite their limited therapeutic efficacy and side effects on patients^28^. In addition to monomer compounds, herbal remedies applied topically or orally as alternative and complementary therapies for the treatment or prevention of OA are gaining popularity^29^.

According to some research, Chinese herbal medicines contain anti-inflammatory and pain-relieving properties, which has received a lot of attention due to its low side-effects^30^. PDL is a well-known anti-inflammatory formulation and has been used to treat many diseases of chronic and acute inflammation for many years in China^22^. It consists of four major herbs, including *Taraxacum mongolicum* Hand.-Mazz. (Pugongying in Chinese), *Scutellaria baicalensis* Georgi (Huangqin), *Corydalis bungeana* Turcz. (Kudiding), and *Radix Isatidis* (Banlangen). Previous studies established LC–MS methods for the simultaneous determination of baicalin, oroxindin, and corynoline in plasma in rats, and pharmacokinetic parameters indicated that baicalin, oroxindin, and corynoline in PDL have certain drug-like properties^31^. However, whether PDL possesses protective effects against OA is unknown. In this study, we found that PDL retarded the progression of OA in a MIA-induced OA model. PDL may also be able to reverse LPS-induced cellular inflammation by inhibiting the ERK/Akt pathway.

As important immune cells, macrophages serve as the first line of defense against invaders (e.g. bacteria, viruses, and fungi)^32^. LPS, also known as endotoxin, is a gram-negative bacteria outer membrane component that is a potent inducer of inflammation and is commonly used to induce inflammation^33^. When macrophages are stimulated by LPS, various inflammatory cytokines are released, such as IL-6, IL-1β, and TNF-α^33,34^.

Pro-inflammatory cytokines are important mediators of the disordered processes involved in the pathophysiology of OA^35^. Most inflammatory molecules are released by the inflamed synovium. The breakdown of the articular cartilage matrix is regulated simultaneously by IL-6, IL-1β, and TNF-α, which together forms a vicious cycle that makes such cytokines a crucial target for OA treatment^36^. TNF-α and IL-1β generation in the joint is regulated by chondrocytes, osteoblasts, synovium-forming cells, and monocytes that have previously been present in the joint or have infiltrated in the joint cavity during the inflammatory response^12^. Researchers found that IL-1β and TNF-α levels are raised in the synovial fluid, synovial membrane, cartilage, and subchondral bone layer of OA patients^37^. Besides, inflammatory joint typically produces IL-6 in response to IL-1β and TNF-α, with the majority of the work being done by chondrocytes, osteoblasts, fibroblast-like synoviocytes, macrophages, and adipocytes^12^. Secreted cytokines interact with relevant factors that lead to the activation of mitogen-activated protein kinase (MAPK) pathways^38^. OA is characterized by degenerative changes in the articular cartilage, including extracellular matrix breakdown and chondrocyte mortality, both of which have been linked to the Akt pathway^39^.

Our findings showed that PDL inhibited LPS-induced inflammatory responses in vitro. Considering the validity of the in vitro experiments, we then conducted in vivo studies in OA mice, measuring mechanical hypersensitivity, serum levels of inflammatory cytokines and mediators, knee cartilaginous tissue mRNA expression levels of inflammation-related genes, histopathological analysis, and radiological examination. In MIA-induced OA mice, we demonstrated that PDL rebounded 50% paw withdrawal threshold related to the joint pain. Histological analysis revealed that the PDL-treated group significantly reduced bone resorption, inflammatory cell infiltration, and cartilage degeneration in the joints. According to radiological examination, the femoral and tibial structures in the mice treated with PDL appeared to be largely unaltered. Based on the results of ELISA and qPCR, PDL inhibited the inflammatory response and lowered the levels of inflammatory factors in serum and mRNA in joint. These findings imply that PDL inhibits the inflammatory mediators and cytokines production of IL-1β, IL-6, and TNF-α in MIA-induced OA mice. Taken together, PDL inhibits the inflammatory response and guards against cartilage degeneration in the joint, slowing the onset and progression of OA.

With the findings obtained from our experiments, it is necessary to point out the limitations concerning animal model and interpretation of the derived results as well. Firstly, although MIA-induced OA does not fully mimic the process of primary OA in humans, the cartilage histological changes, namely, synovitis and cartilage degeneration, are similar^17–19^. It's unclear what kind of OA is caused by injecting MIA into the mouse's knee joint. Thus, more OA models are needed to validate the pharmacological property of PDL against OA in vivo. Secondly, we created an inflammatory model in vitro using LPS-stimulated macrophages. In order to further elucidate the pathology of OA, it is indispensable to take into account stimulating RAW264.7 cells (or chondrocytes) with cytokines involved in the progression of OA in the following studies. Thirdly, we only examined OA-related pain using von Frey filaments. As a result, behavioral evaluation of musculoskeletal pain assessed by weight-bearing and catwalk will be necessary in the future^40,41^. In addition, immunohistochemical staining is needed to confirm changes in the substances that transmit pain in the spinal cord and dorsal root ganglia^42^. Finally, PDL is a complicated formula consisting of four different herbs. Active components in PDL that have potent effect on OA remain to be elucidated and more researches are needed for a better understanding of PDL’s pharmacological effects and the mechanisms of OA itself.

## Conclusion

In the scope of our study, our findings demonstrate that PDL relieves joint pain, suppresses the generation of pro-inflammatory cytokines and mediators, and preserves cartilage in the OA mice model. PDL also shows anti-inflammatory effects in LPS-treated RAW264.7 cells by decreasing the production of TNF-α, IL-1β, and IL-6 via the ERK/Akt signaling pathway. As a result, the findings of this study provide evidence that PDL could be a candidate for the clinical treatment of OA.

## Materials and methods

### Ethics declarations

All animal procedures were reviewed and approved by the Institutional Animal Care and Use Committee (IACUC) at Anhui University of Chinese Medicine. The research report followed the requirements of ARRIVE guidelines 2.0 (https://arriveguidelines.org/arrive-guidelines).

### Reagents

Pudilan Tablets (Jiren Pharmaceutical Co. Ltd., batch No. 2180712), Monosodium Iodoacetate (BBI, Cat. No. A610336-0005), mouse TNF-α ELISA kit (BOSTER, Lot. No. 241171031101), mouse IL-6 ELISA kit (BOSTER, Lot. No. 13217991115), mouse IL-1β ELISA Kit (BOSTER, Lot. No. 1161475917), Page Ruler (Thermo, Lot. No. 01128566), DMEM (Cytiva, Lot No. AG29853165), ECL Ultra kit (NCM, Cat. No: P10100), and TRNzol Universal Regent (TIANGEN, Lot. No. W9511).

### Chemical profiling of PDL

Using the Agilent 1290 ultra-high performance liquid chromatography system (Agilent Instruments, USA), we identified the major components in PDL. The Agilent TC-C_18_ (4.6 mm 100 mm, 2.7 µm) chromatographic column was used, and the mobile phase was 0.2 percent acetic acid (A)-acetonitrile (B), with a flow rate of 0.5 mL/min and a column temperature of 30 °C. The detective wavelength of 289 nm was employed.

### Animals and grouping

Male ICR mice (7 weeks old, weighing about 25 g) were acquired from the Anhui Medical University Laboratory Animal Center (Hefei, China) and acclimated for a week prior to experiments at the 12 h light/dark cycle laboratory settings. Mice were housed in cages, fed normal chow and water on demand, and kept at constant humidity (45 ± 5%) and temperature (25 ± 1 °C) during the feeding process. All of the mice were housed in the Anhui University of Chinese Medicine facility in compliance with Chinese standards on the use and care of laboratory animals. The Anhui University of Chinese Medicine Ethics Committee authorized all experimental protocols in conformity with current Chinese legislation and policy.

After 7 days of acclimation, the MIA-induced OA mice model was established by adapting previously reported procedures^43^. To induce OA in mice, trim and clean the area surrounding the left knee joint of the animals with 70% alcohol after anesthesia with Zoletil (20 mg/kg, Virbac, France). Except for the sham treatment (Sham) group, 0.75 mg of MIA in 10 μl sterile saline (0.9% NaCl) was injected into the left knee joints of all subjects. The dosage was determined in compliance with the report that 0.75 mg of MIA produced referred pain^43,44^. Mice in the sham group were injected with saline into their knee joints accordingly. Afterward, 50 mice in total were randomly assigned to 5 groups, each with 10 mice: (1) Sham treatment group (Sham, treated with 1% carboxymethylcellulose sodium solution), (2) MIA-induced OA group (MIA, treated with 1% carboxymethylcellulose sodium solution), (3) PDL high dose group (treated with PDL, 4 g/kg), (4) PDL medium dose group (treated with PDL, 2 g/kg), (5) Indomethacin group (Indo, treated with indomethacin, 150 mg/kg). The vehicle used in each group is 1% carboxymethylcellulose sodium dissolved in water. Regarding the doses used for all the drugs, we followed the dose conversion guidance between animals and humans as summarized by Nair AB et al*.*^45^, HED (mg/kg) = Animal does (mg/kg) × Km ratio (HED: human equivalent dose, Km: correction factor). The mice in each group were orally administered 1% carboxymethylcellulose sodium solution, PDL or indomethacin 0.2 mL/day for 4 weeks. Mice were gavaged with corresponding drugs once daily following the day of MIA injection until the end of the protocol. 28 days later, mice were euthanized, and the knee joints and serum were extracted for further analysis.

### Measurement of mechanical hypersensitivity

A week before the test, the mice were brought to the behavioral testing laboratory and permitted to adjust unrestrainedly in a cubicle with a wire grid. Before the actual experiment, for the purpose of acclimation, reducing stress and movement during test period, mice were trained for two days by applying von Frey hairs to the plantar surface of the hind paw until the fibers bend. During testing, 0.02, 0.04, 0.07, 0.16, 0.4, 0.6, 1.0, and 2.0 g of fiber were employed. Holding each hair for three seconds until the paw retracts is a positive indicator. O was marked for no response, while X was marked for withdrawal response, starting at a stimulus intensity of 0.16 g. Subsequently, apply force in ascending order up to 2 g or in descending order down to 0.02 when a cutoff response is noticed. Hair was applied in the “up and down” method until a sequence of six responses (e.g., OXOXOX) was obtained. Changes in mechanical pain thresholds in the paws of the OA model were assessed regularly in the weeks after MIA injection.

### Histological preparation

After anesthesia with Zoletil, the knee joints of mice were dissected, and the entire knee joint, including the patella and joint capsule, was collected by cutting the left hind knee joint with orthopedic scissors to remove excess muscle and tissue. After 24 h of fixation in paraformaldehyde, the specimens were decalcified with EDTA decalcification solution, and the decalcification solution was substituted with fresh one every 2 days. After the decalcification procedure was completed, the specimens were dehydrated with different concentrations of gradient ethanol and embedded in paraffin on the front side. The knee was excised towards the sagittal plane to assess the cartilage of the femur and tibia, and here decalcified paraffin-embedded specimens in the sagittal plane were prepared according to the OARSI recommendations described by Glasson et al*.*^46^. Paraffin-embedded tissue Sects. (5 µm thick) spaced 200 µm apart were stained with H&E and Safranin O, respectively, to assess the severity of cartilage damage. The sections were then sequentially dehydrated in different gradients of ethanol concentrations (70%, 80%, 90% and 100%)^47^. Finally, the sections were then transparently immersed in xylene, sealed with neutral resin and dried at room temperature. The histologically representative section was selected for observation under the light microscope (YIB-510, Yueshi Corporation, China) and imaged using a digital camera.

### Histological analyses

To elucidate the histopathological changes in the tibiofemoral articular cartilage of the medial tibiofemoral joint that occur in OA, we quantitatively assessed these changes using the OA cartilage histopathology assessment system (OARSI score, Table 1)^10^ and modified Mankin score (Table 2)^48^. The OARSI score was developed by Pritzker et al. and consists of 6 grades and 4 stages, with a total score ranging from 0 to 24, depending on the severity of OA and cartilage involvement. The higher the value, the more severe the cartilage degeneration^46,47,49,50^. Histological assessment of synovitis was scored by the established method of Krenn et al*.*^51^. This scoring system has the following three subcategories: enlargement of the synovial cell layer (score 0–3), density of resident cells (score 0–3), and inflammatory infiltration (score 0–3). When the scores are summed, a full score of 9 indicates severe synovitis, and a lower score indicates less severe synovitis.

**Table 1:** OARSI histological assessments of osteoarthritis.

Grade	Osteoarthritic damage
0	Normal
0.5	Loss of Safranin-O without structural changes
1	Small fibrillations without loss of cartilage
2	Vertical clefts down to the layer immediately below the superficial layer and some loss of surface lamina
3	Verical clefts/erosion to the calcified cartilage extending to < 25% of the articular surface
4	Vertical clefts/erosion to the calcified cartilage extending to 25–50% of the articular surface
5	Vertical clefts/erosion to the calcified cartilage extending to 50–75% of the articular surface
6	Vertical clefts/erosion to the calcified cartilage extending > 75% of the articular surface

**Table 2:** Modified mankin histology scores.

Category	Subcategory	Score
Structure	Normal	0
	Surface irregularities	1
	Pannus and surface irregularities	2
	Clefts to the transitional zone	3
	Clefts to radial layer	4
	Clefts to calcified zone	5
	Complete disorganization	6
Arrangement of chondrocytes	Normal	0
	Diffuse hypercellularity	1
	Cloning	2
	Hypocellularity	3
Safranin O staining	Normal	0
	Slight reduction	1
	Moderate reduction	2
	Severe reduction	3
	No dye noted	4
Tidemark integrity	Intact	0
	Crossed by blood vessels	1

Histological scores for both scoring systems were assessed and determined by two blinded and trained independent observers (ZZ and CY). Intra- and inter-observer agreement was quantified using intra-class correlation coefficients^17^ (ICCs), which were calculated based on a two-way analysis of variance (ANOVA) and had excellent inter-class correlation coefficients for intra- and inter-assessor reliability with 95% confidence intervals. OARSI scores: 0.92 (0.88–0.95) and 0.88 (0.79–0.94), respectively, modified Mankin score: 0.92 (0.85–0.97) and 0.95 (0.9–0.98).

### X-ray radiographic assessment

Four weeks after administration, the animals were anesthetized and placed in the prone position, and X-rays were taken with a high-frequency automatic animal X-ray system (HF100HA, Mikasa X-ray Co. Ltd., Japan). To correctly compare hind legs, mice were radiographed under equal exposure settings. Standardized radiographs of the mouse's whole skeleton were obtained at 46 kV in manual mode with an exposure time of 2.37 mAs. X-ray images were used to visualize the knee joint gap, bone contours, bone formation, and osteosclerosis in mice. Two independent researchers were asked to analyze the photographs blindly, and score them on the Kellgren-Lawrence (K-L) grading scale. The K-L classification^52^ was used to determine the degree of knee degeneration (Table 3).

**Table 3:** Kellgren–Lawrence classification of osteoarthritis.

Grade	Description	
0	Normal	No radiological findings of osteoarthritis
1	Questionable	Doubtful narrowing of joint space and possible osteophytic lipping
2	Mild	Definite osteophytes and possible narrowing of joint space
3	Moderate	Moderate multiple osteophytes, definite narrowing of joint space, small pseudocystic areas with sclerotic walls and possible deformity of bone contour
4	Severe	Large osteophytes, marked narrowing of joint space, severe sclerosis and definite deformity of bone contour

### qPCR analysis

The expression of *IL-1β*, *IL-6*, and *TNF-α* mRNA in tibiofemoral joint was detected by qPCR. The cartilage from the tibia and femur of mice was dissected with modified methods by Zheng et al.^53^. Specifically, a dissecting microscope or magnifying lamp was used to help identify the "cap" of white cartilage. Once identified, the hind limb of mice was hold and carefully peel off with forceps. Then, the cartilage was snap-frozen and stored at -80℃ for further analysis. The 50 mg of frozen cartilage tissue was ground into powder in liquid nitrogen to make it homogenized. Total RNA was extracted from knee cartilage with TRNzol. After chloroform was added and mixed, the solution was centrifuged at 12,000 rpm for 10 min at 4 °C, according to the manufacturer’s instructions. For quantitative analysis, 500 ng of total RNA was utilized to perform the reverse transcription reaction with the High-Capacity cDNA Reverse Transcription Kit (Thermo Fisher Scientific, USA). The Roche Light Cycler 480 II (Roche, Germany) was used to assess gene expression using the FastStart DNA Master SYBR Green kit (Roche, Germany). The primer sequences for the qPCR experiments are listed in Table 4.

**Table 4:** Quantitative real time PCR primer sequences.

Gene	Sequence	
*IL-1β*	Forward	5′ACAAGGCTGCCCCGACTAT3′
	Reverse	5′CTCCTGGTATGAAGTGGCAAATC3′
*IL-6*	Forward	5′GCCCACCAAGAACGATAGTCA3′
	Reverse	5′CAAGAAGGCAACTGGATGGAA3′
*TNF-α*	Forward	5′TGGGACAGTGACCTGGACTGT3′
	Reverse	5′TTCGGAAAGCCCATTTGAGT3′
*GAPDH*	Forward	5′GGCCTCCAAGGAGTAAGAAA3′
	Reverse	5′GCCCCTCCTGTTATTATGG3′

### Enzyme-linked immunosorbent assay (ELISA)

After 28 days, the mice’s blood was drawn, allowed to clot at room temperature for more than an hour in the EP tube, centrifuged at 3,000 rpm for 10 min at 4 °C, and the serum was stored in a -20 °C refrigerator. According to the manufacturer's instructions, ELISA was used to detect IL-1β, IL-6 and TNF-α in serum or cell supernatant.

### Cell culture

The murine macrophage RAW264.7 cells were obtained from the National Collection of Authenticated Cell Cultures (Shanghai, China). Cells were cultured in DMEM medium containing 10% fetal bovine serum. They were incubated in the CO_2_ constant-temperature incubator (BluePard, China) at 37 °C with 5% CO_2_. This experiment used log-phase RAW264.7 cells in a strong development stage. The RAW264. 7 cells were divided into groups of blank (Con), model (LPS, 1000 ng/mL LPS), low, medium, and high doses (PDL 50, 100 and 200 μg/mL + 1000 ng/mL LPS). After pretreatment with low, medium, and high concentrations of PDL for 12 h, 1000 ng/mL LPS was added to the model group as well as the PDL low, medium, and high dose groups and cultured for 24 h. The blank group was not treated, and instead an equivalent amount of culture medium was added.

### Cell counting kit 8 (CCK-8) assay

PDL working solutions with concentrations of 50, 100, and 200 μg/mL were prepared in serum-free DMEM medium. In 96-well plates with 100 μL serum-free media in each well, RAW264.7 cells in the logarithmic growth phase were seeded at a density of 1 × 10^4^ cells per well. After 24 h, 10 μL of CCK-8 (Biosharp, China) was added to each well, and after 4 h of incubation at 37 °C, the concentration of each well was determined using the microplate reader (Rayto, China) at 450 nm wavelength (be careful not to create air bubbles in the wells since they can affect the OD value). In this experiment, each concentration included six replicate wells.

### Western blotting analysis

The cells were seeded at a density of 1 × 10^6^ cells/mL in the 6-well plates and incubated for 24 h. The supernatant was removed, washed with PBS, and lysed by adding 200 μL of RIPA lysis buffer before centrifuging at 4 °C for 15 min at 12,000 rpm. The protein content of the supernatant was determined using BCA kits and then denatured for 5 min at 95 °C with 2X Laemmli in a 1:1 volume ratio. Electrophoresis was carried out on the sodium dodecyl sulfate–polyacrylamide gel electrophoresis system (SDS-PAGE), first at 100 V constant voltage for 30 min, then at 150 V constant voltage for 60 min. Following electrophoresis, the film was transferred to a polyvinylidene fluoride (PVDF) membrane (Millipore, USA) and blocked for 60 min at 37 °C with 3% Bovine serum albumin (BSA). After rinsing in PBST, antibodies against Akt, ERK, p-Akt, p-ERK, and GAPDH were added and incubated overnight at 4 °C. The PVDF membrane was rinsed with PBST and incubated with the secondary antibody at 37 °C for 1 h. The PVDF membrane was washed three times with PBST before adding chemiluminescent developer dropwise to the PVDF membrane, which was then covered from light for a few seconds before being developed with a chemiluminescent gel imager (Amershan imger 600, USA).

### Statistical analysis

All data information was tallied using Excel tables. Data analysis was completed using SPSS 23.0 software, and graphs were drawn in GraphPad Prism 8.2. Firstly, we tested whether the distribution was normal or not. For data that conformed to normal distribution, the mean ± standard deviation was described, and the independent samples t-test was used for comparison between groups and the paired samples t-test for comparison within groups. For data that did not conform to normal distribution, the median (interquartile spacing) was described, and the Mann–Whitney U test (Mann–Whitney U) was used for comparison between groups and the Wilcoxon test (Wilcoxon) for comparison within groups. The P value of less than 0.05 is considered statistically significant. NS, not significant, *p < 0.05, **p < 0.01, ***p < 0.005, ****p < 0.0001.

## Supplementary Information

Supplementary Information.

## Data Availability

All data included in this study can be obtained by contacting the corresponding author.
