# Supplementary material for: Protective effects of Pudilan Tablets against osteoarthritis in mice induced by monosodium iodoacetate
Source: Sci Rep. 2023 Feb 16;13:2760. doi: 10.1038/s41598-023-29976-0 (PMC9935914; doi:10.1038/s41598-023-29976-0)
Supplement: Supplementary file 1 — Supplementary Information. [file 41598_2023_29976_MOESM1_ESM.pdf]

Supplementary Information
western blot source data for Figure 8

Figure 8A

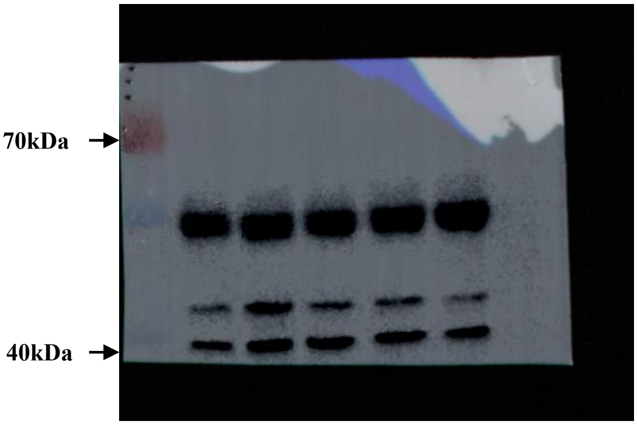

Left blot:
Primary antibody: p-ERK (E-4), (mouse, sc-7383, Santa Cruz)
Secondary antibody: HRP-conjugated Goat Anti-Mouse IgG (#D110087-0100, Sangon Biotech)
Ladder: PageRuler Prestained Protein Ladder(#26616, Thermo Scientific)

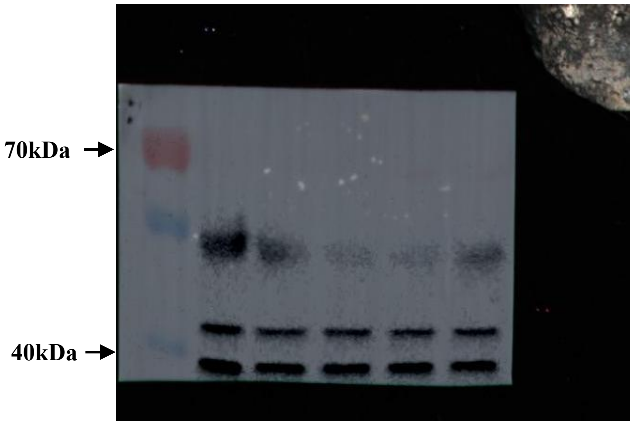

Right blot:
Primary antibody: ERK1/2, (mouse, sc-514302, Santa Cruz)
Secondary antibody: HRP-conjugated Goat Anti-Mouse IgG (#D110087-0100, Sangon Biotech)
Ladder: Ladder: PageRuler Prestained Protein Ladder(#26616, Thermo Scientific)

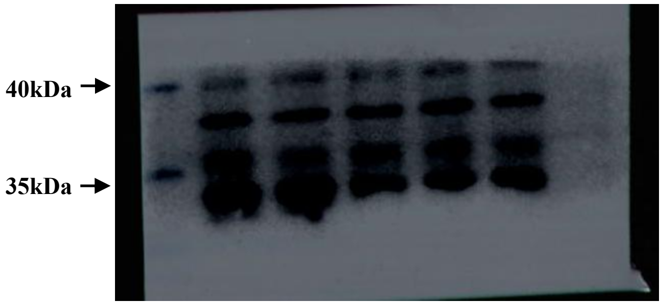

Top blot:
Primary antibody: GAPDH (G-9) (mouse, sc-365062, Santa Cruz)
Secondary antibody: HRP-conjugated Goat Anti-Mouse IgG (#D110087-0100, Sangon Biotech)
Ladder: PageRuler Prestained Protein Ladder(#26616, Thermo Scientific)

Figure 8B

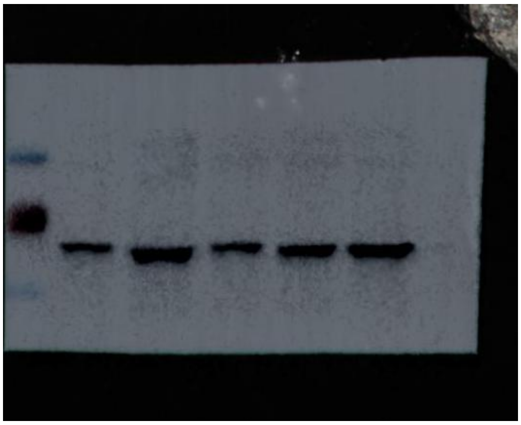

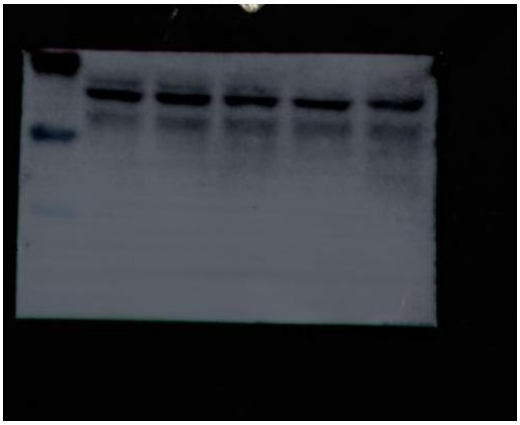

Left blot:
Primary antibody: p-Akt (Ser473) (rabbit, 193H12, Cell Signaling)
Secondary antibody: HRP-conjugated Goat Anti-Rabbit IgG (#D110058-0100, Sangon Biotech)
Ladder: Ladder: PageRuler Prestained Protein Ladder(#26616, Thermo Scientific)

Right blot:
Primary antibody: AKT1 (mouse, BM1612, Boster Biotech)
Secondary antibody: HRP-conjugated Goat Anti-Mouse IgG (#D110087-0100, Sangon Biotech)
Ladder: PageRuler Prestained Protein Ladder(#26616, Thermo Scientific)

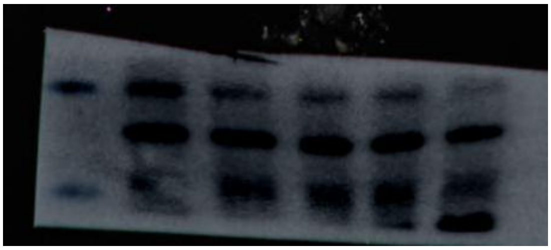

Top blot:
Primary antibody: GAPDH (G-9) (mouse, sc-365062, Santa Cruz)
Secondary antibody: HRP-conjugated Goat Anti-Mouse IgG (#D110087-0100, Sangon Biotech)
Ladder: PageRuler Prestained Protein Ladder(#26616, Thermo Scientific)
